# Chemical Constituents of the Ethyl Acetate Extract of *Belamcanda chinensis *(L.) DC Roots and Their Antitumor Activities

**DOI:** 10.3390/molecules17056156

**Published:** 2012-05-24

**Authors:** Mingchuan Liu, Shengjie Yang, Linhong Jin, Deyu Hu, Zhibing Wu, Song Yang

**Affiliations:** State Key Laboratory Breeding Base of Green Pesticide and Agricultural Bioengineering, Key Laboratory of Green Pesticide and Agricultural Bioengineering, Ministry of Education, Guizhou University, Guiyang 550025, China; Email: gzucrdfc@gmail.com (M.L.); yangsdqj@126.com (S.Y.); linhong_j@126.com (L.J.); gzgdjhzx@126.com (D.H.); wzb1171@163.com (Z.W.)

**Keywords:** *Belamcanda chinensis *(L.) DC, chemical constituents, antitumor activity, cell apoptosis

## Abstract

An activity-directed fractionation and purification process was used to isolate antitumor compounds from the roots of *Belamcanda chinensis *(L.) DC. The ethyl acetate extract showed greater antitumor activities than the other extracts, consequently leading to the isolation of 18 compounds identified as *β-*sitosterol (**1**), dausterol (**2**), quercetin (**3**), kampferol (**4**), shikimic acid (**5**), gallic acid (**6**), ursolic acid (**7**), betulin (**8**), betulonic acid (**9**), betulone (**10**), tectoridin (**11**), irisflorentin (**12**), 4′,5,6-trihydroxy-7-methoxyisoflavone (**13**), tectorigenin (**14**), irilins A (**15**), iridin (**16**), irigenin (**17**), and iristectongenin A (**18**). Compounds **3**–**10**, **13**, and **15** were isolated from *B. chinensis* for the first time. Compounds **4** and **7**–**10** showed potent cytotoxic activities against PC3, MGC-803, Bcap-37, and MCF-7 cell lines. The mechanism of the antitumor action of compound **7** was preliminarily investigated through acridine orange/ethidium bromide (AO/EB) staining, Hoechst 33258 staining, and terminal deoxynucleotidyl transferase dUTP nick end labeling (TUNEL) assay, which indicated the growth inhibition of MGC-803 cells via the induction of tumor cell apoptosis.

## 1. Introduction

*Belamcanda chinensis *(L.) DC from the Iridaceaefamily consists of about 60 genera and 800 species worldwide and mainly two genera (Iridacea and Belamcanda) in China. *B. chinensis *is a shrub that is mainly found distributed in the southwestern part of China, particularly in Guizhou, Yunnan, and Sichuan provinces [1,2]. *B. chinensis* is a perennial herbaceous plant with fan-shaped leaves that reach 2 to 3 feet in length on branching stems. The root of *B. chinensis *is a widely used important medicine in China for curing pulmonary diseases, acute and chronic pharyngitis, asthma, and cancer [3]. The Chinese Pharmacopoeia (2005 edition) listed *B. chinensis *as an official drug, which showed that the plant could be used as an anticancer herb. However, so far the constituents with anticancer activity of the plant remain unclear. Thus it is necessary to identify the potent antitumor compounds from this plant.

Previous chemical investigations on *B. chinensis *had discovered the main chemical components of *B. chinensis*, including isoflavonoids [4,5,6,7] and iridal-type triterpenoids [8,9,10]. We have recently investigated the chemical constituents of *B. chinensis* systematically, and we tested the antitumor activity of the extracts and compounds. The current study was conducted to validate the medicinal use of *B. chinensis*. Finally, the isolation and structural elucidation of 18 compounds from the ethyl acetate extracts, which included eight is of lavones, two flavones, four triterpenes, were performed. Ten of the compounds were isolated from *B. chinensis *for the first time. During the screening for the antitumor agents from tropical medicinal plants, the ethyl acetate extract of *B. chinensis *exhibited great antitumor activity, and some of the compounds isolated from the ethyl acetate extract showed moderate antitumor activity against PC3, MGC-803, Bcap-37, MCF-7 cell lines and less cytotoxic activities toNIH3T3 cells which suggesting that these compounds can be used as lead compounds for further research.

## 2. Results and Discussion

### 2.1. Isolation and Identification

**Figure 1:**
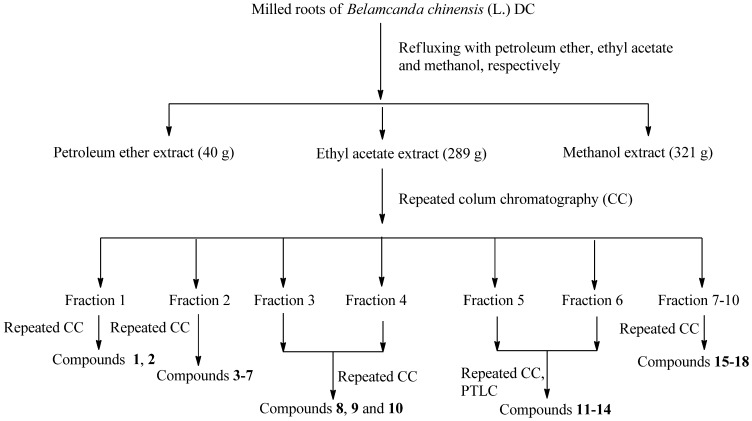
Extraction, fraction, and column chromatography separation of *B. chinensis *roots.

The dried roots of *B. chinensis *(7.5 kg) were refluxed for 6 h with petroleum ether, ethyl acetate, and methanol, respectively. Each solvent extraction process was performed twice, and the extracts were combined and concentrated using a vacuum rotary evaporator at 50 °C to afford the petroleum ether extract (40 g), ethyl acetate extract (289 g), and methanol extract (321 g), respectively, which were evaluated for their antitumor activity using an MTT assay. The results suggest that the antitumor agents were mainly contained in the ethyl acetate extract. Further experiments were performed on the ethyl acetate extract to separate its antitumor compounds. The ethyl acetate extract was subjected to successive chromatographic fractionation and purification to yield compounds **1**–**18**, as shown in Figure 1.

The isolated compounds were identified via spectroscopic analyses, including ^1^H- and^13^C-NMR spectroscopy. The results of the analyses were compared with the NMR and IR data reported in literature to identify the chemical structures of the isolates. Compounds **3**–**10**, **13**, and **15** were isolated from *B. chinensis *for the first time.

### 2.2. Antitumor Activities

The *in vitro* cytotoxic activity of all the compounds was evaluated against PC3, MGC-803, Bcap-37 and NIH3T3 cell lines. Adriamycin (ADM) and the compounds were dissolved using DMSO (negative control). The negative control cells were treated with culture medium containing 0.1% DMSO, while ADM, which showed very high antitumor activity against many kinds of tumor cells, was used as positive control. The inhibitory percentage of cells was treated with 20 μmol/L or 50 μg/mL of each compound or extract for 72 h (Table 1). The results showed that the ethyl acetate extract had greatest antitumor activity and pentacyclic triterpenes (compounds **7**–**10**) from the ethyl acetate extract exhibited moderate inhibitory activities against the growth of human carcinoma cell lines, which are higher than their activities against normal cell line NIH3T3. However the is of lavonoids (compounds **11**–**13**) did not show obvious antitumor activities. The results suggest that the ethyl acetate extract of *B. chinensis *exhibited greater antitumor activities mainly because of the pentacyclic triterpene constituents. At the same time, compared with the crude extracts, the inhibitory activities of the isolated compounds were markedly reduced. The decrease in antitumor activity may be attributed to the synergistic effect of the chemical compounds of the ethyl acetate extract. 

**Table 1:** Antitumor activities of the extracts and isolated compounds on the proliferation of different cancer cell lines.

Test Extracts and Compounds	Inhibitory Rate for Different Cancer Cell Lines (%, mean ± SD) ^a^				
	MGC-803 ^b^	Bcap-37 ^c^	MCF-7 ^c^	PC3 ^d^	NIH3T3 ^e^
Petroleum ether extract ^f^	14.3 ± 9.1	43.1 ± 6.1	33.2 ± 1.1	27.1 ± 5.2	2.6 ± 12.4
Ethyl acetate extract ^f^	94.1 ± 2.8	76.4 ± 4.6	80.4 ± 2.9	86.2 ± 2.1	36.5 ± 7.3
Methanol extract ^f^	51.2 ± 2.1	51.6 ± 5.1	50.0 ± 2.1	65.7 ± 7.8	69.9 ± 4.8
ADM ^g^	92.1 ± 1.3	92.1 ± 1.1	91.1 ± 2.2	93.4 ± 2.6	99.4 ± 0.4
Quercetin (**3**) ^h^	19.2 ± 2.8	41.3 ± 2.9	43.5 ± 6.3	21.8 ± 8.9	5.8 ± 7.7
Kampferol (**4**)	58.2 ± 3.0	51.2 ± 8.1	39.2 ± 6.8	46.1 ± 5.9	11.1 ± 6.7
Ursolic acid (**7**)	51.7 ± 5.6	48.4 ± 5.9	49.4 ± 4.1	57.7 ±1.9	21.7 ± 4.9
Betulin (**8**)	43.7 ± 6.7	53.2 ± 3.2	53.2 ± 5.4	17.3 ± 5.2	33.5 ± 7.1
Betulonic acid (**9**)	68.1 ± 2.6	44.9 ± 2.9	56.1 ± 4.4	52.4 ± 4.2	22.1 ± 6.2
Betulone (**10**)	52.2 ± 5.3	54.2 ± 2.2	64.7 ± 7.3	52.3 ± 3.3	36.3 ± 7.1
Tectoridin (**11**)	12.6 ± 2.6	17.3 ± 4.2	10.0 ± 7.9	18.2 ± 6.2	5.9 ± 5.2
Irisflorentin (**12**)	29.4 ± 4.9	17.0 ± 9.8	39.1 ± 5.0	34.0 ± 4.8	25.0 ± 8.1
4′,5,6-Trihydroxy-7-methoxyisoflavone (**13**)	13.4 ± 7.5	12.9 ± 4.8	20.9 ± 8.8	14.9 ± 2.0	1.7 ± 1.7
Tectorigenin (**14**)	22.6 ± 3.3	18.7 ± 5.4	23.7 ± 4.9	28.1 ± 4.8	18.4 ± 6.1
Irilins A (**15**)	13.4 ± 7.5	12.7 ± 5.1	10.4 ± 3.5	19.3 ± 4.3	9.0 ± 4.8
Iridin (**16**)	12.4 ± 2.7	21.8 ± 3.6	11.8 ± 5.2	22.7 ± 5.2	12.4 ± 7.3
Irigenin (**17**)	25.6 ± 5.3	20.1 ± 7.2	25.1 ± 1.2	25.7 ± 2.2	15.3 ± 1.8
Iristectongenin A (**18**)	26.6 ± 4.9	22.1 ± 8.2	19.1 ± 7.2	25.7 ± 2.2	5.2 ± 6.9

Note: ^a^ Inhibitory percentage of cells treated with 20 μmol/L of each compound for 72 h and SD = standard deviation; ^b^ Stomach cancer; ^c^ Breast cancer; ^d^ Prostate cancer; ^e^ Mouse Fibroblasts; ^f^ Inhibitory percentage of cells treated with 50 μg/mL of each extract for 72 h; ^g^ The standard compound used for comparison of activities; ^h^ Inhibitory percentage of cells treated with 20 µmol/L of each compound for 72 h.

### 2.3. Investigation of Cell Apoptosis

**Figure 2:**
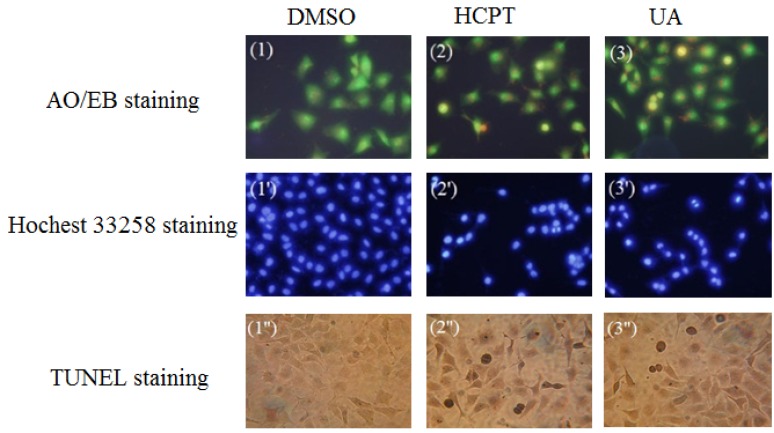
Investigation on the changes in the morphological characteristics of MGC-803 cells during UA-induced apoptosis.

Ursolic acid (UA) was obtained from *B. chinensis* for the first time. UA was also found to have the greatest potency against the growth of human carcinoma cell lines and little cytotoxic effect on NIH3T3 cells among the isolated constituents. The IC_50_ of ursolic acid against MGC-803 cells was determined to be 22.32 ± 2.7 μmol/L using the MTT assay. Therefore, the morphological change in MGC-803 cells treated by UA was investigated through acridine orange/ethidium bromide (AO/EB) staining and Hoechst 33258 staining under fluorescence microscopy to determine whether the growth inhibitory activity of UA is related to the induction of apoptosis. Terminal deoxynucleotidyl transferase dUTP nick end labeling (TUNEL) assay was also conducted to confirm cell apoptosis. Hydroxy camptothecine (HCPT) was used as positive control, and the results are shown in Figure 2.

#### 2.3.1. Acridine Orange/Ethidium Bromide (AO/EB) Staining

AO is a vital dye that could stain nuclear DNA across an intact cell membrane, where as EB could only stain cells that have lost membrane integrity. The stained cells revealed the following four different types under a fluorescence microscope: green coloration of living cells with normal structure; red coloration of non-apoptotic dead cells with normal structure; green coloration of early apoptotic cells with morphous in the form of pycnosis; and orange coloration of late apoptotic cells with morphous in the form of pycnosis. The cytotoxicity of UA at a concentration of 10 μmol/L against MGC-803 cells for 48 h was detected via AO/EB staining, with hydroxyl camptothecine (HCPT) as positive control.

As can be seen in Figure 2(1–3), the amorphous appeared pycnotic after the change in the cells treated with UA for 48 h. Green, live MGC-803 cells with normal morphology were observed in DMSO. The UA displayed green-yellow and orange-yellow dots in MGC-803 cells, showing early and late apoptotic cells for 48 h. In conclusion, the cells presented apoptotic morphous. The appearance of little red cells indicated that the compound UA was associated with low cytotoxicity. Therefore, it can be concluded that UA could induce apoptosis without significant cytotoxicity.

#### 2.3.2. Hoechst 33258 Staining

Live cells with uniformly light blue nuclei were treated with Hoechst 33258 and observed under a fluorescence microscope. The apoptotic cells exhibited bright blue nuclei because of karyopyknosis and chromatin condensation, and the nuclei of dead cells could not be stained. The UA induced apoptosis at 10 μmol/L against MGC-803 cells for 48 h as detected using Hoechst 33258 staining. The HCPT concentration was also 10 μmol/L against the MGC-803 cells for 48 h.

As can be seen in Figure 2(1′–3′), the cells of the negative group were normal blue. Compared with DMSO, the HCPT (positive control) cells appeared compact, condensed, and crescent-shaped, which are the typical apoptosis characteristics. After treatment with UA for 48 h, most cell nuclei appeared to be highly condensed (brightly stained) and crescent-shaped, indicating that UA induced apoptosis against MGC-803 cell lines. These results were identical with the results of the previous AO/EB double staining.

#### 2.3.3. Terminal Deoxynucleotidyl Transferase dUTP Nick End Labeling (TUNEL) Assay

TUNEL is a popular method for identifying apoptotic cells *in situ *via the detection of DNA fragmentation. The cells were observed under a fluorescence microscope, where brown precipitate was the result of positive apoptosis. UA and HCPT were at concentrations of 10 μmol/L against MGC-803 cells for 24 h. As can be seen in Figure 2(1″–3″), the cells of the DMSO did not appear as brown precipitates, whereas the cells of the other groups, namely, UA and HCPT, appeared as brown precipitates. Therefore, it can be concluded that UA induced apoptosis against MGC-803. The results were identical with the results of a previous experiment.

## 3. Experimental Section

### 3.1. General Procedures and Reagents

The melting points of the products were determined using an XT-4 binocular microscope (Beijing Tech Instrument Co. Ltd., Beijing, China). Infrared spectra were recorded on a Bruker VECTOR22 spectrometer in KBr disks. ^1^H-NMR and ^13^C-NMR were recorded using a JEOL-ECX500 spectrometer at 22 °C, with tetramethylsilane as the internal standard and CDCl_3_, DMSO-*d*_6_, CD_3_COCD_3_, or CD_3_OD as the solvent. Column chromatography was performed using silica gel (200–300 meshes) (Qingdao Marine Chemistry Co., Qingdao, China) and silica gel H (Qingdao Marine Chemistry Co., Qingdao, China), Sephadex LH-20 (GE Healthcare Bio-Sciences AB, Uppsala, Sweden), Amberlite XAD-7HP (Rohm & Haas Co., Philly, PA, USA), HP-20 (Mitsubishi Chemical Corp., Toukyu Met, Japan), and MCI-gel CHP 20 P (Mitsubishi Chemical Corp., Toukyu Met, Japan). All other chemicals were of analytical reagent gradeand used without further purification.

### 3.2. Plant Materials

Fresh samples of *B. chinensis *were collected from Bijie, Guizhou Province in China, in August 2011. Prof. Qing-De Long, Department of Medicine, Guiyang Medical University, identified the plant material. A voucher specimen was deposited at Guiyang Medical University, Guiyang, China.

### 3.3. Extraction and Isolation of Antitumor Compounds

The dried roots of *B. chinensis* (7.5 kg) were refluxed with petroleum ether (20 L), ethyl acetate (20 L), and methanol (30 L), respectively (3 times). Petroleum ether extract (40 g), ethyl acetate extract (289 g), and methanol extract (321 g) were obtained. The viscous dark mass of the ethyl acetate extract was adsorbed on silica gel (200–300 mesh) for column chromatography over a silica gel column packed in petroleum ether. The column was eluted successively with petroleum ether, a mixture of petroleum ether, and ethyl acetate (20:1, 15:1, 10:1, 5:1, and 1:1), chloroform, and finally, a mixture of chloroform and methanol (10:1, 5:1, and 1:1). Ten fractions were obtained. Fraction 1 (12 g) was applied to qsilica gel (200–300 mesh) column eluted with petroleum ether–EtOAc (10:1 to 0:1) to yield five fractions. Each fraction was separated via repeated silica gel chromatographyeluted with petroleum ether–EtOAc (10:1 to 0:1) to yield *β*-sitosterol (**1**, 17 mg) anddaucosterol (**2**, 10 mg). Fraction 2 (17 g) was applied to a silica gel (200–300 mesh) column eluted with petroleum ether–EtOAc and CHCl_3_–MeOH (10:1 to 0:1) to yield seven fractions of A–G. Fraction A was applied to asilica gel H columneluted with petroleum ether–EtOAc (20:1 to 0:1) to yield a yellow powder (4 g), which was applied to an Amberlite XAD7HP column first and then to a Sephadex LH-20 column using MeOH as elutant to obtain quercetin (**3**, 10 mg) and kampferol (**4**, 9 mg). Fraction B was separated using asilica gel H column eluted with petroleum ether–EtOAc (20:1 to 0:1) to yield a viscous dark mass (230 mg). Fraction B was then decolorized and separated on MCI geleluted with 50%–90% MeOH-H_2_O to yield shikimic acid (**5**, 13 mg) and gallic acid (**6**, 11 mg). Fractions Cand D were separated using HP-20 resineluted with 50%–90% MeOH–H_2_O, decolorized on Amberlite XAD-7HP first and then on MCI gel, and purified using a Sephadex LH-20 column eluted with MeOH to yield ursolic acid (**7**, 6 mg). Fractions 3 and 4 were applied to asilica gel (200–300 mesh) column eluted with petroleum ether–EtOAc (15:1 to 0:1) and EtOAc–MeOH (10:1 to 0:1) to yield a brown gum (208 mg), and then they were decolorized on MCI gel to yield two fractions, namely, Fractions 1′ and 2′. Fraction 2′ was purified using silica gel (100–200 mesh) eluted with petroleum ether–EtOAc (20:1 to 0:1) to yield betulin (**8**, 7 mg), betulonic acid (**9**, 5 mg), and betulone (**10**, 9 mg). Fractions 5 and 6 were applied to asilica gel (200–300 mesh) column eluted with petroleum ether–EtOAc (10:1 to 0:1) and CHCl_3_–MeOH (20:1 to 0:1) to afford five fractions, namely, Fractions 1″–5″. Fractions 1″–3″ were purified using a Sephadex LH-20 column eluted with MeOH to yield tectoridin (**11**, 24 mg) and irisflorentin (**12**, 120 mg). Fractions 4″ and 5″ were purified using PTLC to yield 4′,5,6-trihydoxy-7-methoxyisoflavone (**13**, 17 mg) and tectorigenin (**14**, 40 mg). Fractions 7–10 were applied to asilica gel (200–300 mesh) column eluted with petroleum ether–EtOAc (15:1 to 0:1) and EtOAc–MeOH (10:1 to 0:1) to yield three fractions, namely, Fraction 1–3′′′. Fraction 3′′′ was separated using silica gel (100–200 mesh) and purified through Sephadex LH-20 to yield irigenin (**17**, 30 mg) and a yellow powder (27 mg), which was consequently separated using MCI gel eluted with MeOH–H_2_O (20%–90%) to yield irilins A (**15**, 4 mg), iridin (**16**, 40 mg), and iristectorigenin A (**18**, 21 mg).

### 3.4. Spectroscopic Data

*Quercetin* (**3**): C_15_H_10_O_7_, yellow powder, mp 306–308 °C, IR (KBr, cm^−1^) *v*: 3346, 1653, 1616, 1506; ^1^H-NMR (CD_3_OD) δ: 7.70 (1H, d, *J *= 2 Hz, H-2′′), 7.62 (1H, dd, *J *= 2, 2.5 Hz, H-6), 6.85 (1H, d, *J* = 2 Hz, H-5′), 6.34 (1H, d, *J *= 2 Hz, H-8), 6.08 (1H, d, *J *= 1.5 Hz, H-6). ^13^C-NMR (CD_3_OD) δ: 175.9 (C-4), 164.1 (C-7), 161.1 (C-5), 156.7 (C-9), 147.4 (C-2), 146.6 (C-3′), 144.5 (C-4′), 122.8 (C-1′), 120.3 (C-6′), 114.7 (C-5′′), 114.6 (C-2′), 103.0 (C-10), 97.8 (C-6), 93.1 (C-8). The above data were identical to the literature data [11].

*Kampferol* (**4**): C_15_H_10_O_6_, yellow powder, mp 281–283 °C. IR (KBr, cm^−^^1^) *v*: 3419, 1653, 1521. ^1^H-NMR (CD_3_OD) δ: 12.05 (1H, s, 5-OH), 8.01 (2H, d, *J *= 7.9 Hz, H-2′, H-6′), 6.92 (2H, d, *J *= 9.2 Hz, H-3′,5′), 6.40 (1H, d, *J *= 2.0 Hz, H-8), 6.14 (1H, d, *J *=2.0 Hz, H-6); ^13^C-NMR (CD_3_OD) δ: 175.3 (C-4), 164.1 (C-7), 161.3 (C-5), 159.2 (C-4′), 146.0 (C-2), 135.8 (C-3), 129.6 (C-2′), 122.4 (C-1′), 115.5 (C-5), 103.3 (C-10), 98.3 (C-6), 93.7 (C-8). The above data were identical to the literature data [12].

*Shikimic acid* (**5**): C_7_H_10_O_5_, white powder, mp 180–182 °C. IR (KBr, cm^−^^1^) *v*: 3383, 1681, 1647, 677, 603. ^1^H-NMR (CD_3_OD) δ: 6.78 (1H, s, 1H), 4.35(1H, s, H-5), 3.97 (1H, q, *J *= 5 Hz, H-4), 3.70 (1H, q, *J *= 10 Hz, H-3), 2.68 (1H, dd, *J *= 10, 5 Hz, H-2), 2.18 (1H, dd, *J *= 10, 5 Hz, H-6). ^13^C-NMR (CD_3_OD) δ: 168.7 (COOH), 137.5 (C-2), 129.3 (C-1), 71.3 (C-4), 67.1 (C-5), 66.0 (C-3), 30.3 (C-6). The above data were identical to the literature data [13].

*Gallic acid* (**6**): C_7_H_10_O_5_, white powder, mp 246–248 °C. IR (KBr, cm^−^^1^) *v*: 3402, 3261, 2362, 1621, 1228, 1033. ^1^H-NMR (CD_3_OD) δ: 7.11 (2H, s, PhH). ^13^C-NMR (CD_3_OD) δ: 170.5 (COOH), 146.3 (C-1), 141.5 (C-2), 105.3 (C-4), 125.6 (C-5). The R_f_ is the same as the standard sample in different solvents and the above data were identical to the literature data [13].

*Ursolic acid* (**7**): C_30_H_48_O_3_, white powder, mp 257–259 °C. IR (KBr, cm^−^^1^) *v*: 3444, 2928, 2868, 1683, 1458, 1028. ^1^H-NMR (CD_3_OD) δ: 0.78 (CH_3_, d, *J *= 10 Hz, H-29), 0.84 (CH_3_, d, *J *= 10 Hz, H-30), 0.90 (CH_3_, s, H-23), 0.92 (CH_3_, s, H-26), 0.97 (CH_3_, s, H-25), 1.09 (CH_3_, s, H-27), 3.20 (1H, d, *J *= 5 Hz, H-3), 5.2 (1H, t, *J *= 1.5 Hz, H-12). ^13^C-NMR (CD_3_OD) δ: 180.1 (C-28), 138.3 (C-13), 125.6 (C-12), 78.1 (C-3), 55.4 (C-5), 53.1 (C-18), 41.9 (C-14), 39.5 (C-8), 39.1 (C-19), 38.9 (C-1), 38.7 (C-4), 38.5 (C-20), 36.8 (C-10), 36.7 (C-22), 33.0 (C-7), 30.4 (C-21), 27.9 (C-23), 27.4 (C-15), 26.6 (C-2), 24.2 (C-16), 23.0 (C-11), 22.7 (C-27), 21.2 (C-30), 18.3 (C-6), 16.5 (C-26), 16.2 (C-29), 14.7 (C-25), 14.6 (C-24). The R_f_ is the same as the standard sample in different solvents and the above data were identical to the literature data [14].

*Betulin* (**8**): C_30_H_50_O_2_, white powder, mp 234–236 °C. IR (KBr, cm^−^^1^) *v*: 3498, 2941, 1456, 1033. ^1^H-NMR (CDCl_3_) δ: 3.79 (1H, d, *J *= 10 Hz, H-3), 4.67 (1H, s, H-29), 4.56 (1H, s, H-29), 3.77 (1H, d, *J *= 5 Hz, H-28), 3.31 (1H, d, *J *= 10 Hz, H-28), 3.18 (1H, d, *J *= 5.5 Hz, H-3), 1.66 (3H, s, H-30). ^13^C-NMR (CDCl_3_) δ: 150.5 (C-20), 109.7 (C-29), 79.0 (C-3), 60.6 (C-28), 55.3 (C-5), 50.4 (C-9), 48.8 (C-18), 47.8 (C-17,19), 42.7 (C-14), 40.9 (C-8), 38.9 (C-4), 38.7 (C-1), 37.4 (C-13), 37.2 (C-10), 34.2 (C-7), 34.0 (C-22), 29.8 (C-21), 29.2 (C-16), 28.0 (C-23), 27.4 (C-2), 27.1 (C-15), 25.3 (C-12), 20.8 (C-11), 19.1 (C-30), 18.4 (C-6), 16.1 (C-25), 16.0 (C-26), 15.4 (C-24), 14.8 (C-27). The above data were identical to the literature data [15].

*Betulonic acid *(**9**): C_30_H_46_O_3_, white powder, mp 291–293 °C. IR (KBr, cm^−^^1^) *v*: 3444, 2947, 1699, 1683. ^1^H-NMR (CDCl_3_) δ: 3.01–2.96 (3H, m, H-2, 19), 4.71 (1H, s, H-29), 4.58 (1H, s, H-29), 1.68 (3H, s, H-30). ^13^C-NMR (CDCl_3_) δ: 218.5 (C-3), 182.4 (C-28), 150.4 (C-20), 109.8 (C-29), 56.4 (C-17), 54.9 (C-5), 49.9 (C-9), 49.2 (C-18), 47.4 (C-4), 47.0 (C-19), 42.5 (C-14), 40.6 (C-8), 39.6 (C-1), 38.5 (C-13), 37.1 (C-22), 36.9 (C-10), 34.1 (C-2), 33.6 (C-7), 32.1 (C-16), 30.6 (C-21), 29.7 (C-15), 26.7 (C-23), 25.5 (C-12), 21.4 (C-11), 21.0 (C-24), 19.6 (C-6), 19.4 (C-30), 16.0 (C-26), 15.8 (C-25), 14.6 (C-27). The above data were identical to the literature data [16].

*Betulone* (**10**): C_30_H_48_O_2_, white powder, mp 201–203 °C. IR (KBr, cm^−^^1^) *v*: 3460, 2945, 2868, 1701, 1458, 1375, 1026. ^1^H-NMR (CDCl_3_) δ: 4.68 (1H, s, H-29a), 4.57 (1H, s, H-29b), 3.80 (1H, d, *J *= 10 Hz, H-28a), 3.34 (1H, d, *J* = 10 Hz, H-28b), 2.47 (2H, m, H-2), 2.40 (2H, m, H-19), 1.95 (1H, m, H-16a), 1.95 (1H, m, H-21a), 1.90 (1H, m, 1a), 1.90 (1H, m, 22a), 1.67 (3H, s, H-30), 1.42 (1H, m, H-21b), 1.38 (1H, m, H-1b), 1.24 (1H, m, H-16b), 1.06 (3H, s, H-23), 1.05 (3H, s, H-26), 1.01 (3H, s, H-24), 0.98 (3H, s, H-27), 0.91 (3H, s, H-25). ^13^C-NMR (CDCl_3_) δ: 218.5 (C-3), 150.4 (C-20), 109.8 (C-19), 60.6 (C-28), 54.9 (C-5), 49.8 (C-9), 48.7 (C-18), 47.8 (C-17,19), 47.4 (C-4), 42.8 (C-14), 40.9 (C-8), 39.6 (C-1), 37.4 (C-13), 36.9 (C-10), 34.2 (C-2), 34.0 (C-22), 33.5 (C-7), 29.7 (C-21), 29.1 (C-16), 27.0 (C-15), 26.7 (C-23), 25.2 (C-12), 21.4 (C-11), 21.1 (C-24), 19.7 (C-6), 19.1 (C-30), 16.0 (C-25), 15.8 (C-26), 14.7 (C-27). The above data were identical to the literature data [17].

*Tectoridin* (**11**): C_16_H_12_O_6_, yellow powder, mp 273–274 °C. IR (KBr, cm^−^^1^) *v*: 3367, 1646, 1635, 1080. ^1^H-NMR (DMSO-*d_6_*) δ: 12.91 (1H, s, 5-OH), 9.58 (1H, s, 4′-OH), 8.41 (1H, s, 2-H), 7.36 (2H, d, *J* = 10 Hz, 2′, 6′-H), 6.86 (1H, s, 8-H), 6.79 (2H, d, *J* = 10 Hz, 3′,5′-H), 5.41 (1H, d, *J* = 5 Hz, 1″-H), 5.13 (2H, d, *J* = 5 Hz, 2″-CH_2_OH), 3.73 (3H, s, 6-OCH_3_), 3.68–3.65 (2H, m, 6″-H), 3.43–3.40 (1H, m, 5″-H), 3.28–3.24 (2H, m, 2″,3″-H), 3.16–3.12 (1H, m, 4″-H). ^13^C-NMR (DMSO-*d_6_*) δ: 181.3 (C-4), 158.0 (C-4′), 157.1 (C-7), 155.2 (C-2), 153.4 (C-5), 153.0 (C-9), 132.9 (C-6), 130.7 (C-2′,6′), 122.5 (C-1′), 121.5 (C-3), 115.6 (C-3′,5′), 106.9 (C-10), 100.6 (Glc-1″), 94.5 (C-8), 77.8 (C-5″), 77.2 (C-3″), 73.6 (C-2″), 70.1 (C-4″), 61.1 (C-6″), 60.8 (6-OCH_3_). The above data were identical to the literature data [18].

*Irisflorentin* (**12**): C_20_H_18_O_8_, white needle, mp 163–165 °C. IR (KBr, cm^−^^1^) *v*: 3462, 1653, 1627, 1583, 1055. ^1^H-NMR (CD_3_COCD_3_) δ: 8.13 (1H, s, 2-H), 6.90 (2H, s, 2′,6′-H), 6.80 (1H, s, 8-H), 6.18 (2H, s, 6,7-OCH_2_O), 3.97 (3H, s, 5-OCH_3_), 3.85 (6H, s, 3′,5′-H), 3.7 (3H, s, 4′-H). ^13^C NMR (CD_3_COCD_3_) δ: 173.9 (C-4), 154.4 (C-5), 153.1 (C-3′), 153.1 (C-5′), 153.0 (C-9), 151.5 (C-2), 141.4 (C-7), 138.2 (C-4′), 136.3 (C-6), 127.9 (C-1′), 125.0 (C-3), 113.9 (C-10), 106.9 (C-2′), 106.9 (C-6′), 102.7 (6,7-OCH_2_O), 93.3 (C-8), 60.5 (5-OCH_3_), 59.7 (4′-OCH_3_), 55.6 (5′-OCH_3_), 55.6 (3′-OCH_3_). The above data were identical to the literature data [18].

*4′,5,6-Trihydroxy-7-methoxyisoflavone* (**13**): C_16_H_12_O_6_, yellow powder, mp 281–283 °C, IR (KBr, cm^−^^1^) *v*: 3460, 1670, 1609, 1448, 1062. ^1^H-NMR (CD_3_OD) δ: 7.89 (1H, s, H-2), 7.31 (2H, d, *J* = 10 Hz, H-2′, 6′), 6.81 (2H, d, *J* = 10 Hz, H-3′, 5′), 6.26 (1H, s, H-8), 3.80 (3H, s, 7-OCH_3_). ^13^C-NMR (CD_3_OD) δ: 180.5 (C-4), 162.8 (C-7), 157.3 (C-4′), 154.3 (C-9), 152.9 (C-2′), 152.7 (C-5), 132.9 (C-6), 130.1 (C-2′,6′), 122.4 (C-3), 122.3 (C-1′), 114.9 (C-3′,5′), 103.5 (C-10), 95.0 (C-8), 59.4(7-OCH_3_). The above data were identical to the literature data [19].

*Tectorigenin* (**14**): C_16_H_12_O_6_, yellow powder, mp 229–231 °C. IR (KBr, cm^−^^1^) *v*: 3479, 1639, 1627, 1508, 1062. ^1^H-NMR (CD_3_OD) δ: 8.07 (1H, s, H-2), 7.35 (2H, d, *J* = 8 Hz, H-2′, 6′), 6.82 (2H, d, *J* = 7.5 Hz, H-3′, 5′), 6.41 (1H, s, H-8), 3.85 (3H, s, 6-OCH_3_). ^13^C-NMR (CD_3_OD) δ: 181.3 (C-4), 157.6 (C-4′), 157.5 (C-7), 153.7 (C-9), 153.6 (C-2), 153.2 (C-5), 131.5 (C-6), 130.0 (C-2′,6′), 122.8 (C-3), 121.9 (C-1′), 114.9 (C-3′,5′), 105.3 (C-10), 93.6 (C-8), 59.0 (6-OCH_3_). The above data were identical to the literature data [20].

*Irilins A* (**15**): C_17_H_14_O_6_, yellow powder, mp 254–257 °C. IR (KBr, cm^−^^1^) *v*: 3421, 1639, 1610, 1580, 1064, 842, 813. ^1^H-NMR (CD_3_OD) δ: 8.04 (1H, s, 2-H), 7.35 (1H, d, *J* = 5 Hz, 6′-H), 6.41 (1H, s, 8-H), 3.84 (3H, s, 6-OCH_3_), 3.79 (3H, s, 7-OCH_3_). ^13^C-NMR (CD_3_OD) δ: 181.2 (C-4), 157.4 (C-2′), 154.2 (C-7), 153.8 (C-2), 153.6 (C-5), 153.2 (C-9), 131.4 (C-6), 130.0 (C-6′), 122.8 (C-4′), 121.9 (C-3), 121.5 (C-1′), 114.5 (C-5′), 109.8 (C-3′), 105.3 (C-10), 93.6 (C-8), 59.5 (6-OCH_3_), 55.1 (7-OCH_3_). The above data were identical to the literature data [21].

*Iridin* (**16**): C_24_H_26_O_12_, yellow powder, mp 210–213 °C. IR (KBr, cm^−^^1^) *v*: 3404, 1649, 1610, 1589, 1047. ^1^H-NMR (CD_3_OD) δ: 8.19 (1H, s, H-2), 6.83 (1H, s, H-8), 6.70 (1H, s, H-2′), 6.69 (1H, d, *J* = 5 Hz, H-6′), 5.08 (1H, d, *J* = 5 Hz, Glc-1′), 3.79 (5′H, s, 4′-OCH_3_), 3.87 (3H, s, 6-OCH_3_), 3.85 (3H, s, 4′-OCH_3_), 3.71–3.37 (m, Glc-H), 3.28 (1H, q, *J* = 3 Hz, H-2″). ^13^C-NMR (CD_3_OD) δ: 181.1 (C-4), 156.6 (C-7), 154.8 (C-2), 153.3 (C-5,9), 153.1 (C-5′), 150.2 (C-3′), 136.6 (C-4′), 132.8 (C-6), 126.5 (C-1′), 123.1 (C-3), 109.8 (C-2′), 107.0 (C-10), 104.6 (C-6′), 100.5 (Glc-1″), 94.1 (C-8), 77.1 (C-3″, 76.6 (C-5″), 73.3 (C-2″), 69.8 (C-4″), 61.1 (C-6″), 60.1 (6-OCH_3_), 59.6 (4′-OCH_3_), 55.1 (5’-OCH_3_). The above data were identical to the literature data [18].

*Irigenin* (**17**): C_16_H_12_O_6_, yellow powder. mp 184–186 °C. IR (KBr, cm^−^^1^) *v*: 3462, 1635, 1579, 1060, 815, 709. ^1^H-NMR (DMSO-*d_6_*) δ: 13.0 (1H, s, 5-OH), 10.75 (1H, s, 7-OH), 9.23 (1H, s, 3′-OH), 8.33 (1H, s, H-2), 6.67 (1H, s, 2′-OH), 6.62 (1H, s, 6′-OH), 6.47 (1H, s, 8-H), 3.75 (3H, s, 5′-OCH_3_), 3.71 (3H, s, 6′-OCH_3_), 3.65 (3H, s, 6-OCH_3_). ^13^C-NMR (DMSO-*d_6_*) δ: 180.8 (C-4), 158.0 (C-5), 155.3 (C-2), 153.8 (C-7), 153.4 (C-9), 153.1 (C-5′), 150.7 (C-3′), 136.8 (C-4′), 131.9 (C-6), 126.6 (C-1′), 122.2 (C-3), 110.8 (C-2′), 105.3 (C-10), 105.0 (C-6′), 94.4 (C-8), 60.5 (6-OCH_3_), 60.4 (4′-OCH_3_), 56.3 (5′-OCH_3_). The above data were identical to the literature data [18].

*Iristectongenin A* (**18**): C_17_H_14_O_7_, yellow powder, mp 232–235 °C. IR (KBr, cm^−^^1^) *v*: 3446, 1653, 1558, 1193, 813, 669. ^1^H-NMR (DMSO-*d_6_*) δ: 13.0 (1H, s, 5-OH), 9.1 (1H, s, 3′-OH), 8.32 (1H, s, H-2), 7.09 (1H, d, *J* = 1.5 Hz, H = 2′), 6.95 (1H, d, *J* = 10 Hz, H-6′), 6.79 (1H, d, *J* = 10 Hz, H-5′), 6.46 (1H, s, H-8), 3.75 (3H, s, 6-OCH_3_), 3.70 (3H, s, 4′-OCH_3_). ^13^C-NMR (DMSO-*d_6_*) δ: 181.0 (C-4), 158.0 (C-9), 154.8 (C-2), 153.8 (C-7), 153.2 (C-5), 147.7 (C-4′), 147.1 (C-3′), 131.9 (C-6), 122.3 (C-1′), 122.2 (C-6), 122.1 (C-3), 115.7 (C-2′), 113.7 (C-5′), 105.3 (C-10), 94.3 (C-8), 60.4 (6-OCH_3_), 56.2 (4′-OCH_3_). The above data were identical to the literature data [22].

### 3.5. Cell Lines and Culture

MGC-803, Bcap-37, MCF-7, PC3 and NIH3T3cell lines were obtained from the Institute of Biochemistry and Cell Biology, China Academy of Science. MGC-803 is stomach cancer cell line, Bcap-37 and MCF-7 are breast cancer cell lines, PC3 is prostate cancer cell line, and NIH3T3 is mouse fibroblast cell line. The entire cancer cell lines were maintained in the RPMI 1640 medium and NIH3T3 was maintained in the DMEM medium. They were supplemented with 10% heat-inactivated fetal bovine serum (FBS) in a humidified atmosphere of 5% CO_2_ at 37 °C.

### 3.6. MTT Assays

All tested compounds were dissolved in DMSO and subsequently diluted in the culture medium prior to the treatment of the cultured cells. The tested cells were plated in 96-well plates at a density 2 × 10^3^ cells/well/100 μL of the proper culture medium and treated with the compounds at 1 μmol/L to 20 μmol/L for 72 h. In parallel, the cells treated with 0.1% DMSO served as negative controls, and the ADM served as positive control. Finally, 100 μL of MTT (Beyotime Co., Jiangsu, China) was added, and the cells were incubated for 4 h. The MTT-formazan formed by metabolically viable cells was dissolved in 100 μL of SDS for 12 h. The absorbance was then measured at 595 nm using a microplate reader (BIO-RAD, model 680), which is directly proportional to the number of living cells in culture [23]. The percentage cytotoxicity was calculated as follows:


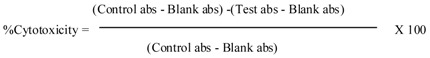


### 3.7. AO/EB Staining

Thecells were seeded at a concentration of 5 × 10^4^ cell/mL in a volume of 0.6 mL on a sterile cover slip in 6-well tissue culture plates. Following incubation, the medium was removed and replaced with fresh medium plus 10% FBS and then supplemented with compounds (10 μmol/L). After the treatment period, the cover slip with monolayer cells was inverted on the glass slide with 20 μL of AO/EB (Beyotime Co., Shanghai, China) stain (100 μg/mL). The fluorescence was read using an IX71SIF-3 fluorescence microscope (OLYMPUS Co., Toukyu Met, Japan).

### 3.8. Hoechst 33258 Staining

The cells grown on the sterile cover slip in 6-well tissue culture plates were treated with compounds for a certain range of treatment time. The culture medium containing compounds was removed, and the cells were fixed in 4% paraformaldehyde for 10 min. The cells were washed twice with PBS, and were consequently stained with 0.5 mL of Hoechst 33258 staining (Beyotime Co., Jiangsu, China) for 5 min. The stained nuclei were washed twice with PBS, and were consequently observed under an IX71SIF-3 fluorescence microscope at 350 nm excitation and 460 nm emissions.

### 3.9. TUNEL Assay

TUNEL assays were performed using a colorimetric TUNEL apoptosis assay kit according to the manufacturer’s instructions (Beyotime). The cells grown in 6-well culture clusters were treated as mentioned in mitochondrial depolarization assay. TheMGC-803 and Bcap-37 cells grown in 6-well tissue culture plates were washed with PBS and fixed in 4% paraformaldehyde for 40 min. The cells were washed once with PBS, and were consequently permeabilized with immunol staining wash buffer (Beyotime) for 2 min on ice. The cells were rewashed once with PBS, and were consequently incubated in 0.3% H_2_O_2_ in methanol at room temperature for 20 min to inactivate the endogenous peroxidases, after which the cells were washed thrice with PBS. Thereafter, the cells were incubated with 2 μL of TdT-enzyme and 48 μL of Biotin-dUTP per specimen for 60 min at 37 °C. The cells were terminated for 10 min, and were consequently incubated with streptavidin-HRP (50 μL per specimen) conjugate diluted at 1:50 in sample diluents for 30 min. The cells were washed three times with PBS, and were consequently incubated with diaminobenzidine solution (200 μL per specimen) for 10 min. Thereafter, the cells were rewashed twice with PBS, and were consequently imaged under an XDS-1B inverted biological microscope (Chongqing Photoelectric Devices Co. Chongqing, China).

### 3.10. Statistical Analysis

All statistical analyses were performed using SPSS 10, and the data were analyzed using one-way ANOVA. The mean separations were performed using the least significant difference method. Each experiment was performed in triplicate, and all experiments were run thrice and yielded similar results. Measurements from all the replicates were combined, and the treatment effects were analyzed.

## 4. Conclusions

Using the bioassay-guided technique, the ethyl acetate extract of the roots of *B. chinensis *was shown to exhibit greater antitumor activities compared with the other two extracts, leading to the isolation of 18 compounds from this extract. The compounds were identified as *β-*sitosterol (**1**), dausterol (**2**), quercetin (**3**), kampferol (**4**), shikimic acid (**5**), gallic acid (**6**), ursolic acid (**7**), betulin (**8**), betulonic acid (**9**), betulone (**10**), tectoridin (**11**), irisflorentin (**12**), 4′,5,6-trihydroxy-7-methoxyisoflavone (**13**), tectorigenin (**14**), irilins A (**15**), iridin (**16**), irigenin (**17**), and iristectongenin A (**18**). Compounds **3**–**10**, **13**, and **15** were isolated from *B. chinensis* for the first time. All compounds were tested for their antitumor activities and pentacyclic triterpenes (compounds **7**–**10**) from the ethyl acetate extract exhibited moderate antiproliferative activities against human carcinoma cell lines. The cell apoptosis-inducing activity of ursolic acid was then further investigated against MGC-803 cell line via AO/EB staining, Hoechst 33258 staining, and TUNEL assay. The results showed that ursolic acid can induce cell apoptosis of MGC-803 cells. The studies of chemical modification of ursolic acid and antitumor bioassay of UA derivatives are currently underway.
